# Genetic analysis of *F8* mutations in five hemophilia a carriers

**DOI:** 10.3389/fmed.2026.1805568

**Published:** 2026-05-15

**Authors:** Huizi Sun, Libin Mei, Xuemei He, Haijie Gao, Xianjing Huang, Jiayan Chen, Meijiao Cai, Ping Li, Yunsheng Ge, Yanru Huang

**Affiliations:** 1 Department of Central Laboratory, Fujian Key Clinical Specialty of Laboratory Medicine, Department of Obstetrics and Gynecology, Women and Children's Hospital, School of Medicine, Xiamen University, Xiamen, Fujian, China; 2 Department of Reproductive Medicine, Department of Obstetrics and Gynecology, Women and Children's Hospital, School of Medicine, Xiamen University, Xiamen, Fujian, China; 3 Xiamen Key Laboratory of Reproduction and Genetics, Women and Children's Hospital, School of Medicine, Xiamen University, Xiamen, Fujian, China; 4 School of Public Health, Xiamen University, Xiamen, Fujian, China

**Keywords:** carrier screening, copy-number variation, F8 gene, gene variant, hemophilia A

## Abstract

**Objectives:**

Hemophilia A (HA) is an X-linked recessive bleeding disorder caused by mutations in the *F8* gene, which exhibits complex molecular mechanisms and high genetic heterogeneity. This study aimed to perform carrier screening and genetic analysis in five phenotypically normal females to identify *F8* variants and assess their implications for genetic counseling and prenatal diagnosis.

**Methods:**

Genomic DNA was extracted from peripheral blood samples. High-throughput sequencing was used to screen for mutations in 455 genes associated with genetic diseases. Long-range PCR (LR-PCR) was employed to detect the inversion of introns 1 and 22 of the *F8* gene. Sanger sequencing validated small deletions. To clarify the complex variations of Subject 4, qPCR, CNV-seq and SV-seq analyses were conducted on her affected uncle.

**Results:**

Subject 1 carried an intron 22 inversion (Inv22) of the *F8* gene. Subjects 2 and 4 carried intron 1 inversions (Inv1). Subject 3 had a small deletion (c.3168_3187del), and Subject 5 had a frameshift deletion (c.4379delA). Notably, Subject 4 was found to carry a rare complex structural variant involving Inv1. QPCR suggested potential duplications in the corresponding genomic region of Subject 4’s uncle. SV-seq identified two duplications on the X chromosome in her uncle, which may disrupt *F8* gene function.

**Conclusion:**

All five subjects carried pathogenic *F8* gene variants, and one affected individual had a pathogenic variant of the gene. Although carriers are asymptomatic, they can transmit the mutant allele to their offspring. Molecular genetics in carriers is crucial for improving genetic screening, prenatal diagnosis, and the development of targeted therapies for HA. This study further enriches the mutation spectrum of the *F8* gene and underscores the importance of comprehensively detecting inversions, copy number variations, and structural rearrangements in the molecular diagnosis of hemophilia A.

## 1 Introduction

Hemophilia A (HA) is the most common inherited bleeding disorder, caused by variants in the factor VIII gene (*F8*) that lead to abnormal production or function of factor VIII (FVIII) protein (1). The causative gene, located on Xq28, follows an X-linked recessive pattern. In this model, female heterozygotes are typically asymptomatic carriers who can transmit the mutant allele to their offspring, while hemizygous males express the phenotype, with an incidence of approximately 1 in 5,000 males (2). The clinical severity of HA is classified into three categories based on plasma factor VIII clotting activity (FVIII: C): severe (FVIII: C < 1%), moderate (FVIII: C 1–5%), and mild (FVIII: C > 5–40%) (3).

Intron 22 inversion (Inv22), a major cause of severe HA, results from homologous recombination between a sequence within intron 22 (*Int22h-1*) and one of two extragenic homologous sequences (*Int22h-2* or *Int22h-3*). This inversion disrupts the *F8* gene structure (4). The intron 1 inversion (Inv1) is another common cause of severe HA (5), arising from recombination between the *int1h-1* sequence in intron 1 and its telomeric homolog *int1h-2*, located approximately 125 kb away, thereby severing the *F8* gene (6). The recombination severs the *F8* gene, thereby preventing the synthesis of functional coagulation factor VIII and ultimately causing severe hemophilia A (6). Inv22 and Inv1 account for approximately 45% and 1–5% of all severe HA cases, respectively (7), with Inv1 identified in 2.94% of the Chinese severe HA population (8). Large *F8* gene duplications are relatively rare, representing about 0.07% of all variants, in contrast to smaller genetic alterations (9).

Although carriers are asymptomatic, they risk transmitting the mutant allele. Molecular genetic analysis in carriers is therefore essential for enhancing genetic screening, prenatal diagnosis, and targeted therapy development for HA. This study further enriches the mutation spectrum of the *F8* gene and highlights the importance of carrier screening and comprehensive molecular diagnosis of hemophilia A.

## 2 Materials and methods

### 2.1 Sample collection

Five phenotypically normal subjects without a personal history of hemophilia or related bleeding disorders were enrolled. Subjects 1–5 participated in carrier screening as part of a preventive genetic counseling program in the Women and Children’s Hospital, Xiamen University, China (Xiamen, China). Subject 4 has a family history of HA, and her paternal uncle (Subject 6) and father were diagnosed with hemophilia A in an external hospital. Both her uncle and father suffered severe bleeding from minor injuries multiple times, and her father died from cerebral hemorrhage. The clinical characteristics and testing items of all subjects are listed in Table 1. Following pre-test genetic counseling, 2 mL of peripheral venous blood was collected from each subject into EDTA-K_2_ vacuum tubes, gently inverted eight times, and stored at 4 °C.

**Table 1:** Clinical features of subjects with mutations in *F8.*

Characteristics	Subject 1	Subject 2	Subject 3	Subject 4	Subject 5	Subject 6
Sex	Female	Female	Female	Female	Female	Male
Age at assessment(years)	36	33	27	29	26	52
Clinical features	NA	NA	NA	NA	NA	HA
History of treatment	NA	NA	NA	NA	NA	Yes
Testing items						
INR (0.88–1.16)	0.96	1.07	1.00	1.08	1.06	/
PT (9.4–12.5 s)	10.70	11.90	11.10	12.00	11.80	/
APTT (25.1–36.5 s)	32.6	32.3	34.5	40.5 ↑	36.5	/
TT (10.3–16.6 s)	12.3	14.1	13.0	15.0	14.6	/
FIB-C (2–4 g/L)	3.54	3.66	2.92	2.65	2.75	/
DD (0–500 ng/mL)	225.00	90.00	47.00	/	185.08	/
FDP (0–5 ug/ml)	/	0.8	/	/	/	/
AT-IIIA (83–128%)	/	/	95.00	/	/	/
PC (70.0–140.0%)	/	/	100.8	107	85.1	/
PS (63.5–149%)	/	/	89.0	23 **↓**	74.0	/
FII: C (70.0–120.0)	/	/	/	120.1 ↑	/	/
FX: C (70.0–120.0)	/	/	/	/	73.1	/
FXII: C (70.0–150.0)	/	/	103.4	48.8 **↓**	30.0 **↓**	/
FV: C (70.0–120.0)	/	/	/	55.7 **↓**	63.4 **↓**	/
FVII: C (70–120)	/	/	/	/	81.6	/

INR, international normalized ratio; PT, prothrombin time; APTT, activated partial thromboplastin time; TT, thrombin time; FIB-C, fibrinogen concentration; FDP, fibrinogen degradation products; AT-IIIA, antithrombin III activity; PC, protein C activity; PS, protein S activity; FII: C, factor II coagulant activity; FX: C, factor X coagulation activity; FXII: C, factor XII coagulant activity; FV: C, factor V coagulation activity; FVII: C, factor VII coagulation activity; The values in brackets are the normal reference ranges of each index. INR, PT, APTT, TT, FIB-C, PS, FII: C, FX: C, FXII: C, FV: C and FVII: C were measured by the clotting assay. DD and FDP were measured by the immunoturbidimetric assay. PC was measured by the chromogenic substrate assay.

### 2.2 Genomic DNA extraction

Genomic DNA was extracted from the peripheral blood of participants using the QIAamp Blood DNA Kit (QIAGEN, Hilden, Germany). DNA purity was assessed with a Nanodrop spectrophotometer (A260/A280 ratios between 1.8 and 2.0), concentration was determined using a Qubit fluorometer (>70 ng/μL), and integrity was verified by 1% agarose gel electrophoresis.

### 2.3 High-throughput sequencing

Libraries were constructed to capture the entire exonic regions and flanking 20 bp sequences of 455 genes associated with 457 genetic diseases at Becon Medical Laboratory. High-throughput sequencing was subsequently performed. After raw data quality control, reads were aligned to the UCSC hg19 reference genome using BWA, and PCR duplicates were removed. Base quality score recalibration and joint genotyping of single-nucleotide variants (SNVs) and insertions/deletions (INDELs) were performed using the Genome Analysis Toolkit (GATK).

### 2.4 Sanger sequencing validation

Primers were designed using Oligo 6 software^1^ to amplify the genomic regions encompassing the *F8* (NM_000132.4) c.3168_3187del and c.4379delA variants. PCR products were sequenced on an ABI 3130xl genetic analyzer (Applied Biosystems; Thermo Fisher Scientific) for validation.

### 2.5 Combined long-range PCR and multiplex PCR for Inv1 and Inv22

**Figure 1:**
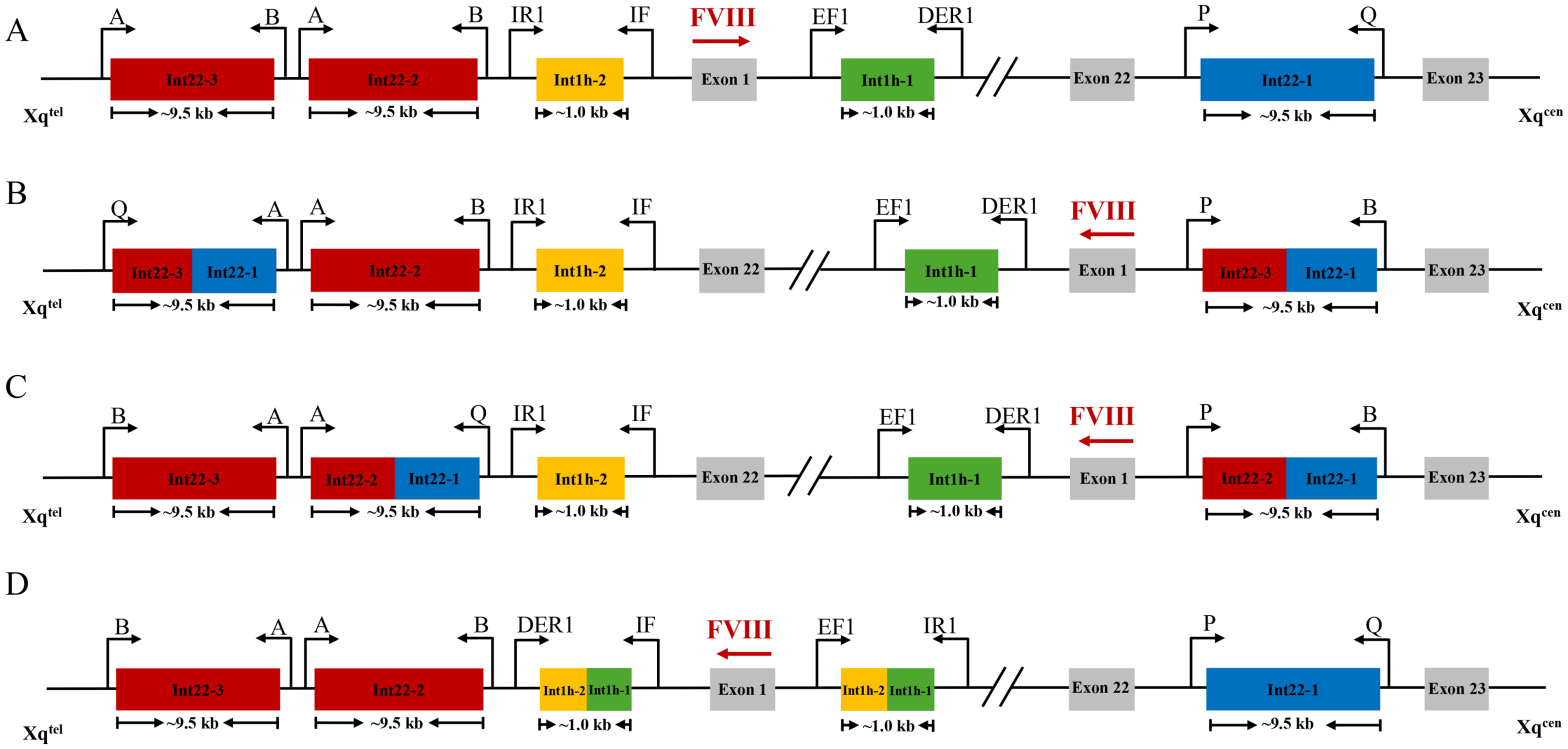
Schematic diagram of the *F8* Inv22 and Inv1 inversion detection. **(A)** The normal structure of the F8 gene. Red and blue boxes represent intron 22 homologous regions (int22h-distal, int22h-2; proximal, and int22h-1; intragenic), yellow and green boxes indicate intron 1 homologous regions (int1h-2; distal and int1h-1; intragenic), and gray boxes represent exon regions. The upper labels indicate primer binding sites (A, B, IR1, IF, EF1, DER1, P, Q). Xq^tel^: X-chromosome q arm telomere, Xq^cen^: X-chromosome q arm centromere. **(B)** Intron 22 type 1 inversion (Inv22 I): homologous recombination occurs between int22h-1 and int22h-3, resulting in fusion of int22h-3 and int22h-1 at both recombination junctions. Exons 1 to 22 are displaced toward the telomere and oriented opposite to their normal orientation. **(C)** Intron 22 type 2 inversion (Inv22 II): homologous recombination occurs between int22h-1 and int22h-2, resulting in fusion of int22h-2 and int22h-1 at both recombination junctions. The FVIII promoter region is displaced toward the centromere. **(D)** Intron 1 inversion (Inv1): homologous recombination occurs between int1h-1 and int1h-2, resulting in fusion of int1h-2 and int1h-1 at both recombination junctions. Exon 1 and the FVIII promoter region are displaced toward the telomere. The illustration is a simplified model of gene structure, with the scale adjusted to not represent the actual sequence length. The actual size shall be based on the number of bases indicated in the figure.

The intron 1 and intron 22 inversions of the *F8* gene were detected using a combined LR-PCR and multiplex PCR strategy (Figure 1). The assay was performed using a modified four-pool multiplex PCR. Specific primers (sequences in Supplementary Table 1) targeting the wild-type and rearranged junctions of intron 1 (EF1, IF, DER1, IR1; 10 μM each) and intron 22 (NP, NQ, P, Q, A, B; 20 μM each) were combined into four primer pools at the ratios specified in Table 2. Each 25 μL PCR reaction was assembled with the components listed in Table 3, containing 50 ng of genomic DNA. After gentle vortexing and centrifugation, amplification was performed on a Bio-Rad T100 thermal cycler under these conditions: initial denaturation at 94 °C for 2 min; 10 cycles of 98 °C for 10 s and 70 °C for 6 min; 20 cycles of 98 °C for 10 s and 68 °C for 6 min; final hold at 4 °C. Subsequently, 4 μL of each PCR product was mixed with 1 μL of 10 × loading buffer and analyzed by electrophoresis on a 0.5% agarose gel in 1 × TAE buffer at 200 V for 30 min, using the DL15000 DNA ladder (Takara) as a size standard. Gels were stained with a nucleic acid dye, and amplicons were visualized under UV light using a GelDoc-It TS imaging system (UVP).

**Table 2:** Multiplex PCR primer-pool composition and pooling ratios.

Primer pool	Multiplex primer panel	Pooling volume ratio
Pooling1	EF1 + DER1 + NP + NQ	1:1:2:2
Pooling2	IF+IR1 + A + B	1:1:2:2
Pooling3	EF1 + IR1 + P + B	1:1:2:2
Pooling4	IF+DER1 + A + Q	1:1:2:2

**Table 3:** Multiplex PCR reaction mixture.

Reagent	Volume
KOD Fx neo	0.4 μL
dNTPs Mixture(2 mM)	5.0 μL
2 × PCR buffer for KOD Fx neo	12.5 μL
Pooling1/ Pooling2/ Pooling3/ Pooling4	1.5 μL
gDNA(50 ng)	X μL
Nuclease-Free Water	bring up to 25 μL

### 2.6 Quantitative PCR (qPCR)

To assess potential copy number variation in the *F8* intron 1 region for Subject 6, qPCR was performed using two primer pairs (Supplementary Table 1) targeting ChrX:154182158–154,233,526. Reactions (20 μL) contained 10 μL SYBR Premix Ex Taq II (Takara), 0.2 μM of each forward and reverse primer, 2 μL of 1:5 diluted cDNA, and nuclease-free water. Amplification was conducted on a Bio-Rad CFX96 system: pre-denaturation at 95 °C for 30 s; 40 cycles of 95 °C for 5 s and 61 °C for 30 s; followed by a melt curve analysis from 65 °C to 95 °C with increments of 0.5 °C for 5 s to verify amplification specificity. The 2^(-ΔΔCt) method was used for relative quantification, normalizing to *GAPDH* and *β-actin*. Statistical significance between groups was determined by Student’s t-test or one-way ANOVA, with a *p*-value < 0.05 considered statistically significant.

### 2.7 CNV-seq

To further validate the duplication in the *F8* intron 1 in Subject 6, CNV-seq was performed on Subject 6’s DNA by BGI Clinical Laboratories (ShenZhen) Co., Ltd. Following previously established protocols (10). Briefly, genomic DNA was fragmented by acoustic shearing, and sequencing libraries were constructed through end repair, A-tailing, adapter ligation, and PCR amplification. Library quality was assessed by Qubit fluorometry and AgilentBioanalyzer 2,100 (concentration ≥2 ng/μL, fragment size ~150–300 bp). Qualified libraries then underwent a series of procedural steps, including single-strand separation, circularization, and rolling circle replication, to generate DNA nanoballs. Subsequently, sequencing was conducted using the combinatorial probe-anchor synthesis (cPAS) method on the MGISEQ-2000 sequencer (BGI, Shenzhen, China). Raw data underwent preprocessing steps, such as sequence alignment, deduplication, and GC correction. CNV analysis was then performed using statistical algorithms to derive the results.

### 2.8 SV-seq

To clarify whether there is a cryptic duplication in the *F8* intron 1 in Subject 6, SV-seq was conducted by Shanghai Jingyin Biotechnology Co., Ltd. SV-seq was performed following previously established method (11). Briefly, genomic DNA is extracted from the sample. After DNA fragmentation, sequencing libraries are constructed through end repair, A-tailing, linker ligation, and PCR amplification. Subsequently, sequencing is performed using the Illumina platform (0.1 × −5 × coverage, 36–100 bp read length). The obtained reads are aligned to the human reference genome (GRCh37/hg19 or GRCh38) using standard bioinformatics algorithms. Structural variations (SVs), including deletions, duplications, insertions, inversions, and translocations, were detected through comprehensive analysis of discordant read pairs, split reads, and read depth signatures. SV calling was performed using integrated algorithms, and candidate variants were filtered based on quality scores, read support, and population frequency.

## 3 Results

### 3.1 High-throughput sequencing and sanger validation

**Figure 2:**
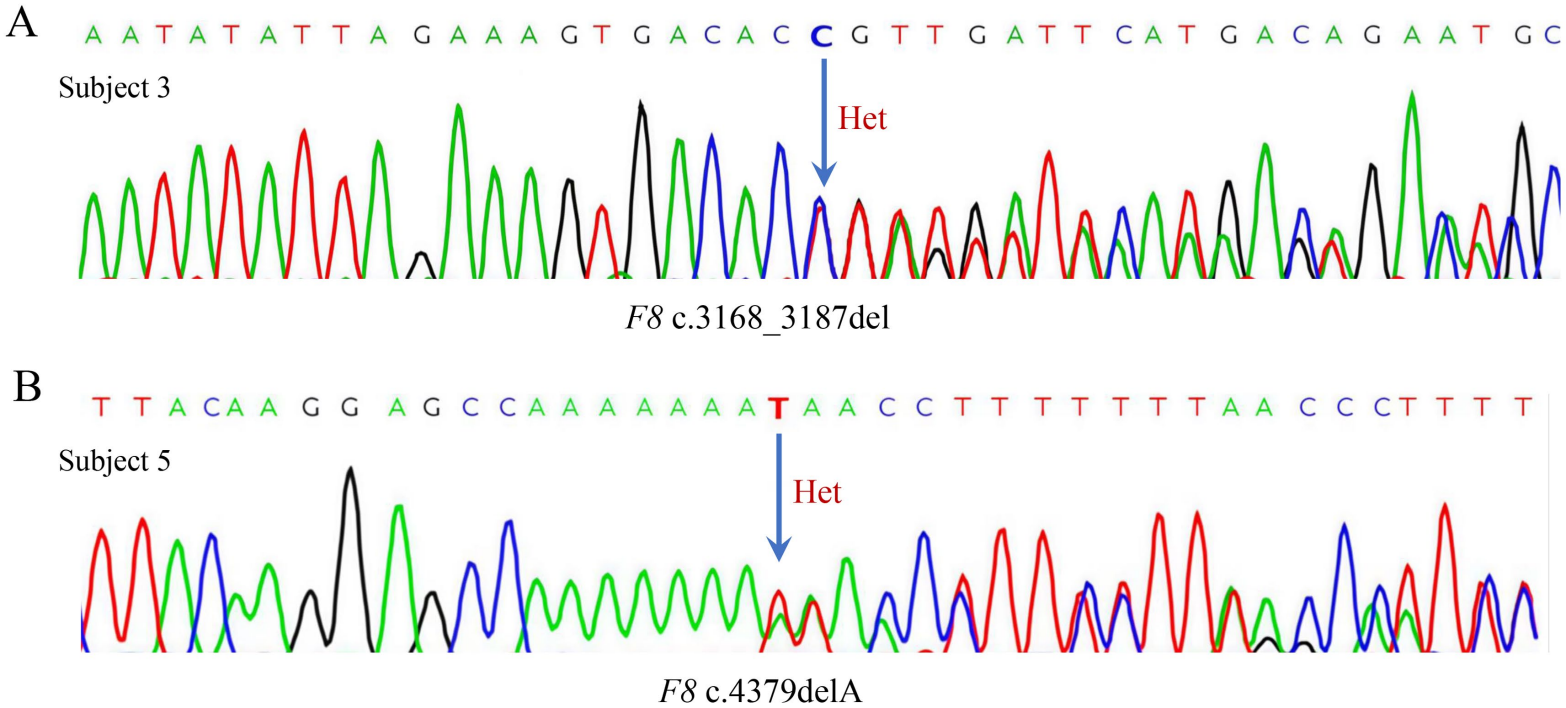
Sanger sequencing results of subject 3 and subject 5. **(A)** Heterozygous *F8* c.3168_3187del variant identified by Sanger sequencing in Subject 3. Arrows denote the mutation sites. **(B)** Heterozygous *F8* c.4379delA variant **identified** by Sanger sequencing in Subject 5. Arrows denote the mutation sites.

High-quality sequencing data (Table 4) were obtained via sequencing. No *F8* point mutations were detected in Subjects 1, 2, and 4. In Subject 3, the c.3168_3187del variant was identified and confirmed by Sanger sequencing (Figure 2A). This variant is not listed in the Human Gene Mutation Database (HGMD) or ClinVar but had been documented in the Leiden Open Variation Database (LOVD) with one literature report (12). Subject 5 carried the c.4379delA variant, which was also confirmed by Sanger sequencing (Figure 2B). The LOVD database^2^ has recorded 41 independent occurrences and recognized this site as a pathogenic variant.

**Table 4:** Quality of high-throughput sequencing data in the target area.

Subject	1	2	3	4	5
Total data (GB)	17.66	16.01	15.03	18.09	13.82
Coverage (%)	99.92	99.91	99.62	99.62	99.60
Specificity (%)	83.13	81.31	81.84	82.87	83.76
Uniformity (%)	98.07	97.59	97.88	98.09	97.47
Proportion of > 30X (%)	98.98	98.13	98.11	98.69	97.62
Average depth (X)	191.33	173.23	161.74	201.77	155.12

### 3.2 LR-PCR analysis

**Figure 3:**
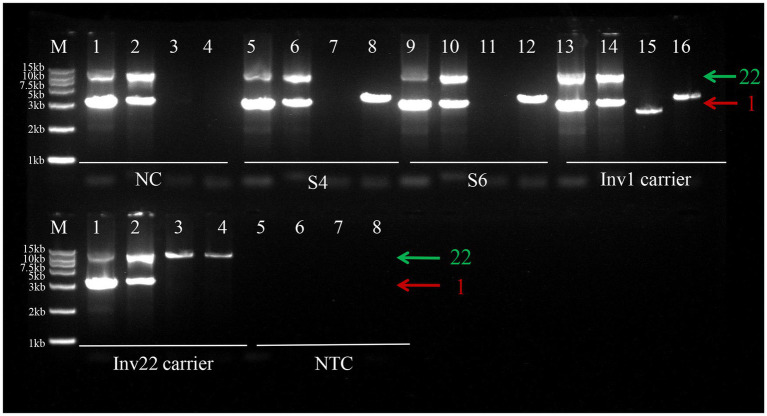
LR-PCR analysis of Subject 4 and Subject 6. Long-range PCR showed no inv22 in Subject 4 (S4) or Subject 6 (S6). An aberrant three-band profile was observed for Inv1 in Subject 4 and Subject 6, inconsistent with wild-type or the heterozygous carrier state. Line 1, 5, 9, and 13 are amplification products of primers EF1, DER1, NP, and NQ. Lanes 2, 6, 10, and 14 are amplification products of primers IF, IR1, A, and B. Lanes 3, 7, 11, and 15 are amplification products of primers EF1, IR1, P, and B. Lanes 4, 8, 12, and 16 are amplification products of primers IF, DER1, A, and Q. Green arrow: intron 22 primer sets; Red arrow: intron 1 primer sets. NC, normal control (wild-type); S4: subject 4; S6: subject 6; Inv1 carrier: *F8* intron 1 inversion carrier; Inv22 carrier: *F8* intron 22 inversion carrier; NTC, no-template control. M: DL15000 DNA ladder.

Subject 1 was identified as a heterozygous carrier of Inv 22. Subject 2 was identified as a heterozygous carrier of Inv1. Subject 4 showed an atypical three-band electrophoretic pattern for Inv1, differing from the classic carrier profile (Figure 3). Subject 6 (the affected uncle) showed a normal Inv22 pattern but the same atypical three-band pattern for Inv1 as Subject 4. To clarify the underlying genetic mechanism, we performed additional genetic analyses for Subject 6 (Subject 4’s uncle), including qPCR, CNV-seq, and SV-seq.

### 3.3 Quantitative PCR analysis

**Figure 4:**
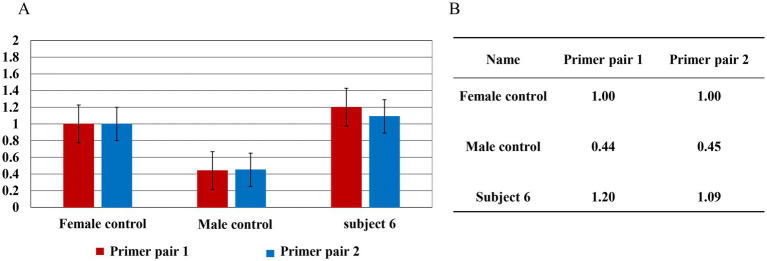
qPCR result for Subject 6. **(A)** Bar graph showing the elevated copy number in Subject 6 relative to female and male controls. **(B)** Relative quantification (RQ) values obtained with two independent primer pairs for the female control, male control, and Subject 6. The data indicate a heterozygous duplication at ChrX:154182158–154,233,526.

QPCR was subsequently performed to validate the duplication at locus ChrX:154182158–154,233,526 in Subject 6. The results demonstrated a significant increase in copy number in the sample from Subject 6 compared to the normal male control, confirming a potential duplication in this genomic region (Figure 4).

### 3.4 CNV-seq validation

CNV-seq identified two CNVs in Subject 6 (Table 5), but no copy number abnormalities were detected on the X chromosome.

**Table 5:** CNV-seq findings for Subject 6.

Variant designation	Classification	Fragment size	Origin of the variant
sseq[GRCh37]8p22p21.3(18,102,071_19263288)x3 chr8:g.18102071 19263288dup	Variant of uncertain significance	1.16 Mb	Unknown
sseq[GRCh37]8p22p22(16,826,647_16977490)x1 chr8:g.16826647 16977490del	Variant of uncertain significance	150.84Kb	Unknown

### 3.5 SV-seq validation

**Figure 5:**
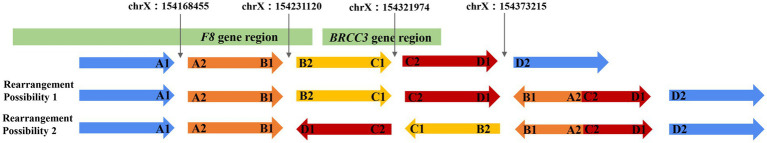
Schematic overview of the SV-seq findings. A1/A2: the upstream and downstream breakpoint ends at chX:154168455. B1/ B2: the upstream and downstream breakpoint ends at chX:154231120. C1/ C2: the upstream and downstream breakpoint ends at chX:154321974. D1/ D2: the upstream and downstream breakpoint ends at chX:154373215.

SV-seq analysis of Subject 6 identified multiple CNVs, including two duplications on the X chromosome: chrX:g.154168455_154231120dup (~63 kb, involving *F8* intron 1 to intron 13) and chrX:g.154321974_154373215dup (Table 6). Based on combined LR-PCR analysis and SV-seq results, two potential structural rearrangement models for the *F8* gene are proposed (Figure 5).

**Table 6:** SV-seq findings for Subject 6.

CNV findings	Variant description	Fragment size	ACMG pathogenicity classification
1 duplication	seq[hg19] dup (8) (p22q21.3) chr8:g.18115980_19235079dup	1.12 Mb	Variant of uncertain significance
2 duplication	seq[hg19] dup(X) (q28) chrX:g.154168455_154231120dup	63 Kb	Variant of uncertain significance
3 duplication	seq[hg19] dup(X) (q28) chrX:g.154321974_154373215dup	51 Kb	Variant of uncertain significance
4 deletion	seq[hg19] del(Y) (q11.223q11.23) chrY:g. 24825000_28025000del(mos 50%)	3.2 Mb	Variant of uncertain significance

## 4 Discussion

The *F8* gene spans approximately 3,186 kb, making it one of the largest genes in its genomic region. It comprises 26 exons and 25 introns, encoding a 2,332 amino acids polypeptide organized in the domain structure A1-A2-B-A3-C1-C2. The A domains are critical for FVIII protein synthesis and activation, while the B domain modulates its secretion. Small deletions or insertions within the B domain can induce frameshift mutations that disrupt normal splicing or transcription (13). The 9.1 kb coding region of *F8* contains 70 CpG dinucleotides, corresponding to 140 potential base-pair change sites. Its high GC content contributes to hypermutability, with approximately 30% of mutations arising *de novo* (14). The extensive allelic heterogeneity and broad spectrum of copy number variations further drive the genetic heterogeneity characteristic of *F8* gene mutations (15). To date, the Human Gene Mutation Database (HGMD) has cataloged 4,412 distinct variants in the *F8* gene,^3^ including inversions, missense and nonsense mutations, deletions, duplications, and insertions/deletions. Among these, inversions represent a pivotal molecular mechanism, accounting for approximately half of all severe HA cases (16). In the present study, all five subjects undergoing carrier screening were found to carry *F8* gene mutations. The detected mutational spectrum included intronic inversions and small nucleotide deletions.

Inv22 in *F8* was detected in Subject 1, while Inv1 was detected in Subject 2 and 4. The Inv22 rearrangement arises from intrachromosomal homologous recombination between the *int22h-1* sequence within *F8* intron 22 and its extragenic homologs located telomeric to the gene (17). This event splits the *F8* gene into two inversely oriented segments: exons 1–22 are translocated to a distal site on Xq in reverse orientation, whereas exons 23–26 remain at the original locus. Consequently, the reading frame is disrupted, preventing the transcription of full-length *F8* mRNA and leading to a severe deficiency of FVIII (18). Inv22 accounts for approximately 45% of all severe HA cases (4). Inv1 occurs at a lower frequency, but it accounts for about 2.94% of HA cases in the Chinese population (8). In patients with Inv1, the inversion results from homologous recombination between the *Int1h-1* sequence in *F8* intron 1 and its telomeric homolog *Int1h-2* (18). This structural rearrangement disrupts the *F8* gene and abrogates the production of full-length mRNA, ultimately leading to the severe HA phenotype due to a complete lack of functional FVIII (19). The detection of these inversions in Subjects 1, 2, and 4 underscores their significant role in the pathogenesis of hemophilia A.

The deletions c.3168_3187del and c.4379delA were identified in the *F8* (NM_000132.4) genes of Subject 3 and Subject 5, respectively. Both variants are located within exon 14, a region characterized by long poly-adenine (poly-A) tracts that confer high susceptibility to replication slippage, making it a well-recognized mutational hotspot for frameshift mutations (20). The c.3168_3187del deletion leads to the substitution of glutamic acid with phenylalanine at amino acid position 1,057 and introduces a premature termination codon four residues downstream, likely resulting in a truncated protein. Although this 20-bp deletion is located within the B-domain, it is close to the A2-domain boundary and may therefore affect A2-A3 domain interactions (1). This variant was first reported in a prenatal diagnosis case in 2009. The male neonate carrying this mutation exhibited typical clinical manifestations of severe HA. Laboratory tests revealed markedly reduced factor VIII clotting activity (FVIII: C) at 0.60% of normal, consistent with the diagnostic criterion for severe HA (FVIII: C < 1%) (12). However, the pathogenic mechanism of this mutation has not yet been experimentally elucidated. The deletions c.4379delA is located within the B-domain of exon 14 in the *F8* gene. Although the B-domain is dispensable for FVIII coagulant activity, it facilitates protein secretion. This deletion mutation results in the substitution of asparagine with isoleucine at amino acid position 1,460 and introduces a premature termination codon five amino acids downstream, likely producing a truncated protein. It has been identified in a Colombian cohort with severe HA, where the carrier exhibited a truncated FVIII protein and severely impaired coagulation activity (21). In a study of the hemophilia population in India, a patient carrying c.4379delA showed reduced FVIII: C (2.6%) and factor VIII antigen levels (FVIII: Ag) below 1%, consistent with a severe HA phenotype (14). The LOVD database lists 41 recorded cases of this mutation, which is classified as pathogenic. The frameshift leads to a premature termination codon (TAG), causing aberrant termination of FVIII synthesis and protein truncation. This truncation prematurely terminates the B-domain of FVIII, impairing its normal interaction with activated factor IX (FIXa) and the tenase complex, and disrupting the coagulation cascade (21). Previous studies have proposed that during *F8* gene replication, slippage events frequently occur in homopolymeric nucleotide tracts, leading to deletion mutations. These events are most prevalent in poly-A regions and are associated with a severe HA phenotype (22). Furthermore, several deletion variants adjacent to this mutation site (including c.4380del, c.4279del, c.4199del, c.4339del, and c.4280del) are also located within the B-domain of exon 14 in the *F8* gene. This domain plays a critical role in FVIII secretion and represents a hotspot for deletion events that frequently lead to frameshift mutations and the development of HA (23, 24).

Subject 4 was found to carry Inv1 with a chromosomal duplication, a complex structural variant rarely reported in HA. Subject 4 married her husband in 2022. Her father and uncles were diagnosed with HA, but none had undergone genetic testing. Due to the family history of hemophilia, the couple sought preimplantation genetic testing (PGT) at the Ethics Board of the Women and Children’s Hospital of Xiamen University, China, to assist in achieving a pregnancy. To elucidate the genetic mechanism of hemophilia in the family, Subject 4 and one of her affected uncles underwent genetic testing. LR-PCR analysis for the *F8* inv22 region yielded normal results for both individuals. However, LR-PCR analysis for *F8* Inv1 revealed an atypical three-band pattern in both Subject 4 and her uncle. Previous literature suggests that such a pattern may correspond to an inversion accompanied by a copy number variation in intron 1 of the *F8* gene (25). It is hypothesized that chromosomal structural abnormalities prevented the amplification of specific fragments, leading to the abnormal banding pattern observed. QPCR analysis of the uncle’s sample indicated a potential duplication in this region. We initially used CNV seq for detection and only found two chromosomal duplication variants on chromosome 8 (Table 3). According to the ClinGen CNV scoring system, the score was 0, indicating that the clinical significance of the variant was unclear. And the genes on these two fragments are not related to HA, so this is not the cause of HA in Subject 6. There are no copy number abnormalities detected on the X chromosome by CNV-seq. Subsequently, SV-seq analysis identified two duplicated copy number variants (CNVs) on the X chromosome in the Subject 6: chrX:g.154168455_154231120dup and chrX:g.154321974_154373215dup. The chrX:g.154168455_154,231,120 duplication is located at Xq28, spanning approximately 63 kb and encompassing *F8*. This gene is classified as triplosensitive in the ClinGen database (https://www.clinicalgenome.org). The *F8* gene is associated with HA, an X-linked recessive disorder characterized clinically by joint and muscle bleeding, frequent cutaneous ecchymoses, and persistent hemorrhage following trauma. The severity of bleeding varies depending on FVIII levels. The chrX:g.154321974_154,373,215 duplication is located at Xq28, spanning approximately 51 kb and encompassing two protein-coding genes, *MTCP1* and *BRCC3*. The *MTCP1* gene may be disrupted. According to OMIM database,^4^ *MTCP1* and *BRCC3* share overlapping critical regions. Loss of *BRCC3* function has been reported to cause a multisystem disorder characterized clinically by moyamoya disease, short stature, hypergonadotropic hypogonadism, facial dysmorphism, dilated cardiomyopathy, and premature hair graying (26) Based on the SV-seq results from the Subject 6, we propose two potential rearrangement scenarios in the *F8* gene (Figure 5). If the rearrangement corresponds to “Possibility 1,” no coding genes would be disrupted, and the two duplications would not result in copy number gains affecting the complete structures of *F8* or *BRCC3*; these CNVs would therefore be considered non-pathogenic. We instead hypothesize that the uncle’s genotype aligns with “Rearrangement Possibility 2,” involving the two CNV duplications (chrX:g.154168455_154231120dup and chrX:g.154321974_154373215dup) accompanied by an inversion at chrX:g.154231120_154,373,215. This complex structural variant is likely disruptive to the *F8* gene at intron 1, leading to loss of gene function. Although SV-seq detected an inversion signal associated with *F8* loss-of-function, the pathogenicity of the two identified duplications has not been previously documented, and their precise mechanistic role remains to be elucidated.

In our study, Subject 6 was found to carry an *F8* Inv1 variant accompanied by a 63 kb duplication, extending from partial int1h-1 sequences into intron 13. This specific structural variant has not been previously reported. *F8* Inv1 variants associated with gene duplications or deletions have been documented in patients with HA. For instance, Sanna et al. (27) reported a patient from Southern Italy with an *F8* Inv1 variant together with a 19.32 kb duplication (extending from partial int1h-1 sequences into intron 6) and a concurrent 41.87 kb extragenic deletion in the telomeric region. You et al. (28) described a Chinese patient with an *F8* Inv1 pattern involving a 227.3 kb duplication and a 2.56 kb deletion, suggesting that mechanisms such as fork stalling and template switching/microhomology-mediated break-induced replication (FoSTeS/MMBIR) and non-allelic homologous recombination (NAHR) may underlie genomic rearrangement. Another HA patient, reported in a 2021 study (29), also carried an *F8* Inv1 with a large duplication and a deletion—the deleted region (chrX: 154235303_154237171) involved int1h-1, while the duplicated region (chrX: 154259274_154376426) involved int1h-2. In our study, the identified *F8* Inv1 with a 63 kb duplication—spanning from partial int1h-1 sequences into intron 13—likely disrupts the transcriptional activity of *F8*, which may explain the hemophilia phenotype observed in Subject 6. The variation discovered in our study is similar to these previously discovered variations. These reports showed that attention should be paid to the combined copy number duplication or deletion of inv1 in F8 gene in the diagnosis of HA. However, not all *F8* inversions with duplications result in a typical HA phenotype. A 2023 study reported an asymptomatic male carring an Inv22 variant with a 0.16 Mb partial duplication (7). In this case, the 210 kb tandem inversion-duplication did not completely abolish *F8* transcriptional activity, as residual wild-type mRNA transcripts were detected. The open reading frame (ORF) remained intact, and the promoter region was unaffected, indicating that the penetrance of *F8* variants may be modulated by multiple factors, including residual transcript levels and the precise architecture of the genomic rearrangement.

Notably, CNV-seq failed to detect the 63 kb and 51 kb duplications in this case due to its restricted resolution at the detection threshold. The standard resolution of CNV-seq is usually 100 kb, although some platforms can achieve accuracy down to 50 kb (30). However, the detection sensitivity of 50–60 kb fragments is significantly reduced when they are located in critical regions (30). SV-seq offers higher detection rate for chromosomal structural variations comparing to traditional methods like CNV-seq (31), especially for cryptic chromosome rearrangements, complex rearrangements, and balanced rearrangements that cause gene destruction (32). However, SV-seq has limitations in detecting highly repetitive regions. In this study, SV-seq did not detect the 150.84 kb deletion on chromosome 8 that was detected by CNV-seq. This may be because the breakpoint of the 150.84 kb deletion is located on the short arm of chromosome 8. There are multiple repetitive sequences in this region. The short read sequencing method cannot detect highly repetitive sequences. So SV-seq may not accurately identify split reads. In a previous report, it was also mentioned that there was a missed detection of SV-seq in the short arm region of chromosome 8 (33). These findings underscore the clinical necessity of integrating multiple complementary methods to ensure accurate genomic characterization.

The limitation of this study is the small sample size, with only one case of complex structural variation identified. Future research should focus on validating verifying the pathogenicity of such variants through expanded cohort screening and functional assays. Another limitation of this study is that the complex rearrangement structure of Subject 6 was inferred only through biological information. Its pathogenicity was not verified by functional experiments. Additionally, due to clinical constraints, including that the subjects refuse to undergo further testing, we were unable to perform RT-PCR to detect abnormal transcripts. Consequently, we could not assess the impact of the rearrangement on RNA splicing and transcription. Moreover, the expression and secretion levels of FVIII protein could not be determined, precluding evaluation of the actual functional consequences of this rearrangement.

To summarize, all five subjects in this study were found to carry pathogenic variants in the *F8* gene. Notably, one affected individual (Subject 6) was identified with a novel *F8* Inv1 variant accompanied by a 63 kb duplication, which has not been previously reported. This finding not only enriches the mutation spectrum of the *F8* gene, but also provides valuable insights for the molecular diagnosis and genetic counseling of HA. Furthermore, the characterization of such complex structural variants offers new perspectives for understanding HA pathogenesis. In clinical practice, for the molecular diagnosis of HA, We need to pay attention to atypical inheritance patterns. When the detection results of LR-PCR exhibit an atypical band pattern, it should be highly suspected that there are complex structural variations, such as inversions accompanied by copy number variations. In the present case of Subject 4 and 6, the F8 Inv1 inversion accompanied by a 63 kb duplication produced a characteristic three-band electrophoretic pattern. Furthermore, the inherent limitations of any single diagnostic modality must be acknowledged. Therefore, we strongly recommend the use of multiple detection methods in combination to improve detection accuracy.

## Data Availability

The datasets presented in this study can be found in online repositories. The names of the repository/repositories and accession number(s) can be found in the article/supplementary material.

## Footnotes

1
http://www.oligo.net/downloads.html

2
https://databases.lovd.nl/shared/genes

3
https://www.hgmd.cf.ac.uk/ac/gene.php?gene=*F8*

4
http://www.omim.org
