# Supplementary material for: Genetic analysis of F8 mutations in five hemophilia a carriers
Source: Front Med (Lausanne). 2026 May 15;13:1805568. doi: 10.3389/fmed.2026.1805568 (PMC13218909; doi:10.3389/fmed.2026.1805568)
Supplement: Supplementary file 1 [file Table_1.DOCX]

Supplementary Table 1

| Primer | Sequence | Amplification region |
| --- | --- | --- |
| F8-EF1 | TTCTACCTGTGCCTGGTTGTGGACC | chrX:154233519-154236411 2893bp |
| F8-DER1 | AGGAGGAAGGGCTGACATTACACAATG |  |
| F8-IF | TTGGATGTGATGTCCTGGTATGCCTC | chrX:154374215+154377276 3062bp |
| F8-IR1 | AGTGTTGGATTGTAAAGGCGAGTGGA |  |
| F8-NP | ACAGGGGAGCAAAGTGAGAAGAGGTT | chrX:154107762-154119510 11749bp |
| F8-NQ | CATTCTGCCTTTCACTTTCAGTGCAATA |  |
| F8-P | GCCCTGCCTGTCCATTACACTGATGACATTATGCTGAC | chrX:154107751-154119821 12071bp |
| F8-Q | GGCCCTACAACCATTCTGCCTTTCACTTTCAGTGCAATA |  |
| F8-A | CACAAGGGGGAAGAGTGTGAGGGTGTGGGATAAGAA | chrX:154615843-154615878;chrX:154684142-154684177 |
| F8-B | CCCCAAACTATAACCAGCACCTTGAACTTACCCTCT | chrX:154693954-154693987；chrX:154606037-154606070 |
| primer pair-1F | ACGCCACCATTACAAAGCAC | chrX:154225170-  154225357 |
| primer pair-1R | TACCATCCAGGCTGAGGTTTA |  |
| primer pair-2F | ACATACCTGGCAGAGACCTGTTTA | chrX:154212956-  154213063 |
| primer pair-2R | CAGAAACAAAGAACTCCTTGATGC |  |
